# QEEG Signatures are Associated with Nonmotor Dysfunctions in Parkinson's Disease and Atypical Parkinsonism: An Integrative Analysis

**DOI:** 10.14336/AD.2022.0514

**Published:** 2023-02-01

**Authors:** Hailing Liu, Zifeng Huang, Bin Deng, Zihan Chang, Xiaohua Yang, Xingfang Guo, Feilan Yuan, Qin Yang, Liming Wang, Haiqiang Zou, Mengyan Li, Zhaohua Zhu, Kunlin Jin, Qing Wang

**Affiliations:** ^1^Department of Neurology, Zhujiang Hospital of Southern Medical University, Guangzhou, Guangdong, China.; ^2^Department of Neurology, Maoming People's Hospital, Maoming, Guangdong, China.; ^3^Department of Neurology, Guangdong Neuroscience Institute, Guangdong General Hospital, Guangdong Academy of Medical Sciences, Guangzhou, China.; ^4^Department of Neurosurgery, General Hospital of Southern Theater Command of PLA, Guangdong, China.; ^5^Department of Neurology, Guangzhou First People's Hospital, School of Medicine, South China University of Technology, Guangzhou, Guangdong, China.; ^6^Clinical Research Centre, Orthopedic Centre, Zhujiang Hospital of Southern Medical University, Guangzhou, Guangdong, China.; ^7^Department of Pharmacology and Neuroscience, University of North Texas Health Science Center, Fort Worth, TX 76107, USA

**Keywords:** Parkinson’s disease, progressive supranuclear palsy, multiple system atrophy, atypical parkinsonism, quantitative electroencephalography, functional connectivity

## Abstract

Parkinson’s disease (PD) and atypical parkinsonism (AP), including progressive supranuclear palsy (PSP) and multiple system atrophy (MSA), share similar nonmotor symptoms. Quantitative electroencephalography (QEEG) can be used to examine the nonmotor symptoms. This study aimed to characterize the patterns of QEEG and functional connectivity (FC) that differentiate PD from PSP or MSA, and explore the correlation between the differential QEEG indices and nonmotor dysfunctions in PD and AP. We enrolled 52 patients with PD, 31 with MSA, 22 with PSP, and 50 age-matched health controls to compare QEEG indices among specific brain regions. One-way analysis of variance was applied to assess QEEG indices between groups; Spearman's correlations were used to examine the relationship between QEEG indices and nonmotor symptoms scale (NMSS) and mini-mental state examination (MMSE). FCs using weighted phase lag index were compared between patients with PD and those with MSA/PSP. Patients with PSP revealed higher scores on the NMSS and lower MMSE scores than those with PD and MSA, with similar disease duration. The delta and theta powers revealed a significant increase in PSP, followed by PD and MSA. Patients with PD presented a significantly lower slow-to-fast ratio than those with PSP in the frontal region, while patients with PD presented significantly higher EEG-slowing indices than patients with MSA. The frontal slow-to-fast ratio showed a negative correlation with MMSE scores in patients with PD and PSP, and a positive correlation with NMSS in the perception and mood domain in patients with PSP but not in those with PD. Compared to PD, MSA presented enhanced FC in theta and delta bands in the posterior region, while PSP revealed decreased FC in the delta band within the frontal-temporal cortex. These findings suggest that QEEG might be a useful tool for evaluating the nonmotor dysfunctions in PD and AP. Our QEEG results suggested that with similar disease duration, the cortical neurodegenerative process was likely exacerbated in patients with PSP, followed by those with PD, and lastly in patients with MSA.

Parkinson's disease (PD) [1, 2], is the second most common neurodegenerative disease worldwide, typically presenting as bradykinesia, rigidity, or tremor, combined with nonmotor dysfunctions resulting from dopamine deficiency. Atypical parkinsonism (AP), such as progressive supranuclear palsy (PSP) and multiple system atrophy (MSA), shares similar symptoms with PD and is easily misdiagnosed as PD, especially in the early stage of the disease due to lacking diagnostic markers [3, 4]. Our previous studies revealed that various markers in PD are related to the severity of disease [1, 5-13]. Nonmotor symptoms, such as cognitive impairment, sleep disorder, and mood disturbance may occur before or after the onset of motor symptoms and affect the patients’ quality of life [12, 14, 15]. Previous studies [16, 17] indicated that nonmotor symptoms may vary in PD and AP. Patients with PSP showed a higher rate of cognitive, behavioral, and urinary dysfunctions, while those with MSA had higher prevalence of urinary dysfunction, sleep/fatigue symptoms, and mood disorders than patients with PD [18, 19].

Electroencephalogram (EEG) [20] is a reliable, noninvasive, and inexpensive diagnostic tool for examining the electrophysiological activities of the brain. Quantitative EEG (QEEG) is a modern EEG analysis method, that involves recording digital EEG signals and using complex mathematical algorithms for data processing, conversion, and analysis [21]. The spectral power of a specific frequency band and EEG-functional connectivity (FC) analysis can be obtained from QEEG. Several lines of evidence have demonstrated that QEEG provides a new technology to quantify cortical synaptic damage or loss in clinical trials of neurodegenerative disease [22]. Several studies[23-26] have suggested that QEEG features provide a valuable variable in distinguishing various etiological dementias, such as dementia with Lewy bodies (DLB), PD with dementia (PDD), and Alzheimer’s disease (AD). A study revealed that QEEG could reflect the amount of phosphorylated α-synuclein in the cortex of patients with PD [27]. Studies have revealed that beta power changes are associated with the severity of motor function, such as dyskinesias and freezing gait [28], while abnormalities in delta or theta band power are related to cognitive deficits in patients with PD [29]. However, QEEG have not been performed to identify differences in nonmotor dysfunctions among various types of Parkinsonisms.

Alterations in the brain FC may precede structural damage and might be a key marker in detecting changes in brain plasticity over short periods in some neurodegenerative diseases [30-32]. PD is characterized by the accumulation of misfolded a-synuclein, leading to the loss of dopaminergic neurons in the substantia nigra, while α-synuclein-immunoreactive inclusions mainly involve oligodendrocytes in MSA. PSP is characterized by the deposition of four repeat tau inclusions in the caudate, midbrain, thalamus, and frontal cortex [33]. Various tools, such as QEEG, functional magnetic resonance imaging (fMRI), and functional near-infrared spectroscopy, have been used to examine brain functional changes. Among all, fMRI has been widely used to study abnormal patterns of FC in PD and AP. In an fMRI study, patients with MSA with predominant parkinsonism (MSA-P) showed greater FC of the posterior cingulate and parietal lobe compared to patients with PD. Relative to healthy controls, lower FC in patients with PSP was widespread and might have preceded grey matter atrophy and was correlated with cognitive decline. In the functional studies, EEG-FC presents the great advantage of studying brain network oscillations at the cortical level with temporal resolution, non-invasively, portably and at a lower cost. However, there is no literature using EEG-FC to explore the differences between PD and AP.

Phase synchronization has been widely used to measure the EEG-FC between different brain regions. Several EEG-FC indices based on phase synchronization have been proposed, including coherence, phase lag index (PLI), and weighted phase lag index (wPLI) [34]. Volume conduction and noise disturbance are considered major obstacles to the effective measurement of phase synchronization [35]. Volume conduction leads to an increase in coherence and phase-locked values. PLI is less affected by phase delay, but sensitive to volume conduction and noise. For wPLI, the observed contribution of phase lead and lag is weighted by the size of the imaginary part of the cross spectrum to reduce sensitivity to noise sources. In conclusion, among the connectivity analyses of EEG and magneto-encephalogram signals, the wPLI is a good candidate parameter for revealing synchronized data [36]. How the application of resting-state EEG-FC and network topology can be applied to examine Parkinsonism presents an interesting topic.

The present study aimed to characterize the patterns of QEEG and EEG-FC that can differentiate patients with PD from those with PSP and MSA. We sought to visualize the differences in resting-state EEG-FC and network topology between PD and MSA/PSP. In addition, correlations between the differential QEEG indices and nonmotor dysfunctions among PD, PSP, and MSA were measured. The potential differences in QEEG indices between PD and MSA/PSP may provide insight into the neurophysiology of the nonmotor dysfunctions that differ between PD and AP.

## MATERIALS AND METHODS

### Participants and standard protocol approval

In the current study, patients who fulfilled the following criteria were enrolled: hospitalized in the Zhujiang Hospital of Southern Medical University between 2017 and 2021. The final diagnosis of PD, MSA, and PSP was determined by two neurologists according to the updated clinical guidelines[37-40]. The exclusion criteria were as follows: (1) diagnosis of DLB, cortical basal ganglia, or uncertain diagnosis; (2) patients who were on acetylcholinesterase inhibitors; (3) patients with PD and a history of cerebrovascular disease, head tumor, or injury; (4) patients with PD and physical diseases, including liver or kidney disease, tumors, or other critical diseases; (5) patients with PD and major psychiatric disorders; and (6) patients with incomplete data or artificial EEG. Health controls (HCs) were recruited from the Medical Examination Centre of Zhujiang Hospital of Southern Medical University. All participants signed an informed consent form.

We recruited a total of 52 patients with PD, 31 with MSA, and 22 with PSP in the present study. Fifty age- and sex-matched HC participants were enrolled. All participants with PD completed standard assessment measures as follows: an appropriate demographic form, including duration, and levodopa equivalent daily dose (LEDD). The Unified Parkinson's Disease Rating Scale III (UPDRS-III) and H&Y stage subscales were administered to assess the severity of motor dysfunction. The severity of nonmotor dysfunction was assessed with the Mini-Mental State Examination (MMSE), nonmotor symptoms scale (NMSS), Hamilton Anxiety Scale (HAMA), and Hamilton Depression Scale (HAMD).

This study was approved by the Ethics Committee of Zhujiang Hospital of Southern Medical University (NO: 2021-KY-110-02) and complied with the ethical standards of the 1975 Declaration of Helsinki and the 1999 National Institutes of Health Policy and Guidelines for Human Subjects conduct.

### EEG recording and analysis

Resting EEG refers to a continuous EEG signal lasting 20 min in a dark and quiet room. According to the 10-20 International System, 19 electrodes were placed on all participants with their eyes closed when they were relaxed and awake. The electrodes included Fp1, Fp2, F3, F4, Fz, F7, F8, C3, C4, Cz, P3, P4, Pz, T3, T4, T5, T6, O1, and O2.

### Quantitative EEG analysis

EEGLAB toolbox version 13 was used to preprocess resting EEG recordings. The data of the Fp1 and Fp2 electrodes were not calculated due to artifact contamination related to eye movement. A 1Hz high-pass filter and 40Hz low-pass filter were used to clear the EEG signal, and a notch filter at 50 Hz was applied. The recording was then divided into 2-second periods, and any remaining artifacts were checked. The EEG with artifacts was removed. The number of epochs available for analysis varied, ranging from 1-3 min. Fast Fourier Transform was used to analyze the absolute spectral power (ASP) of resting EEG data. ASP for the following common frequency bands was conducted: delta (1-4 Hz), theta (4-8 Hz), alpha (8-13 Hz), and beta bands (13-30 Hz). The slow-to-fast frequency ration (SFR) was subsequently calculated, using the formula [(delta + theta)/ (alpha + beta)]. In addition, the alpha/theta ratio of the enrolled patients was determined. The SFR and alpha/theta ratio could then be computed in global and specific brain regions, including the frontal (F3, F4), central (C3, C4), temporal (T3, T4, T5, T6), parietal (P3, P4), and occipital areas. The global ratio was calculated based on the average of all electrodes.

### Functional connectivity analysis

EEG-FC analysis was applied to EEG data using wPLI. EEG electrodes were defined as network nodes, and wPLI values between electrode pairs were defined as edges to construct a topology network. The false discovery rate (FDR) measurement provided by the network-based statistic connectome MATLAB package and HERMES toolbox was used to address the problems of multiple testing and perform graph theory analysis. Pairwise comparisons were conducted by Student’s t-test, and FDR-corrected P < 0.05 was considered statistically significant.

### Statistical analysis

SPSS 24.0 was used to perform statistical analyses. Analysis of variance (ANOVA) and chi-square tests, followed by Bonferroni analysis, were performed to compare characteristics of the sample population and QEEG indices among patients with PD, MSA, PSP, and HCs for normal data. Kruskal-Wallis H analysis was used for nonnormal data. ASP was logarithmically transformed to achieve homogeneity of variance for normally distributed data. Spearman's correlations were used to explore the relationship between QEEG indices and clinical scales in PD, MSA, and PSP. Logistic regressions were applied to confirm whether QEEG indices could be differential factors between PD and PSP/MSA after adjusting for sex, age, LEDD, MMSE, UPDRS-III, NMSS, HAMA, and HAMD scores. To assess the diagnostic value of different QEEG indices in the identification of PD and MSA and the discrimination from PD with PSP, receiver operating characteristic (ROC) analysis was conducted as a diagnosis tool. A probability value of less than 0.05 was considered significant.

**Figure 1.**
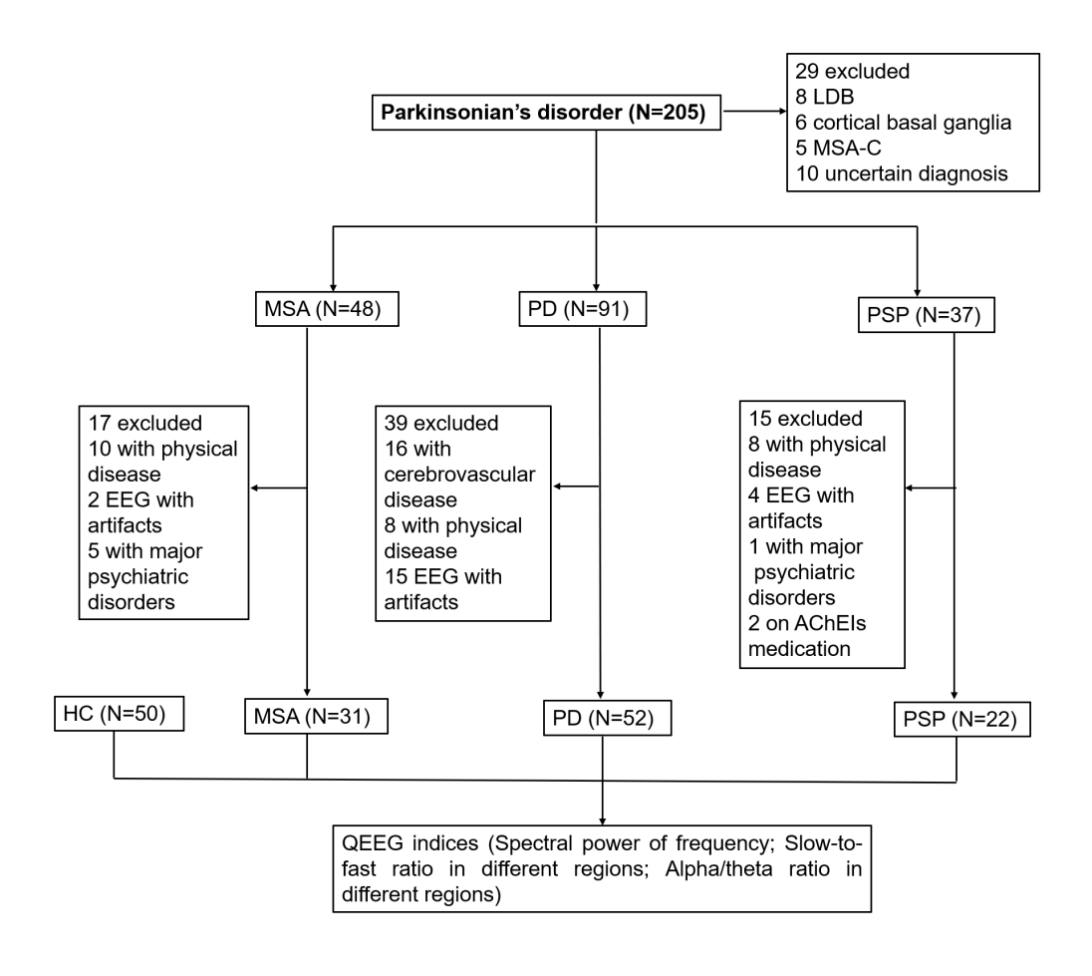
The study flow diagram.

## RESULTS

### Demographic characteristics and clinical manifestations of selected patients

In total, 205 patients with Parkinsonian symptoms were retrospectively reviewed. Twenty-nine patients with uncertain diagnoses of probable MSA with cerebellar ataxia (MSA-C), DLB, and CBD were excluded, and 71 patients were ultimately excluded due to excessive artifacts or meeting the exclusion criteria. Consequently, only 52 patients with PD, 31 with MSA, and 22 with PSP and 50 HC participants were enrolled in the present study. The study flow diagram is shown in Figure 1. The percentage of men in each group was as follows: PD 80%, MSA 68%, PSP 81%, and HC 68%. The mean ages of the groups were 62.0±9.7, 60.2±7.5, 64.5±7.5, and 63.9±8.6 years, respectively. The durations of the PD, MSA, and PSP groups were 3.5±3.6, 2.4±1.6, and 3.1±2.4 years, respectively. The differences in sex, age, and duration were not significant. Clinical characteristics, such as UPDRS-III and H&Y stage and NMSS scores, in the attention/memory and perception domains, were significantly higher, and MMSE scores were lower in patients with PSP than in those with MSA and PD. Cardiovascular domain and HAMA scores were significantly higher in patients with MSA than in those with PD and PSP, while the HAMD scores were significantly higher in patients with PSP than in those with PD and MSA. The demographic and clinical characteristics of the different groups are described in Table 1.

### Comparison of spectral power among PD, MSA, PSP and HCs

**Figure 2.**
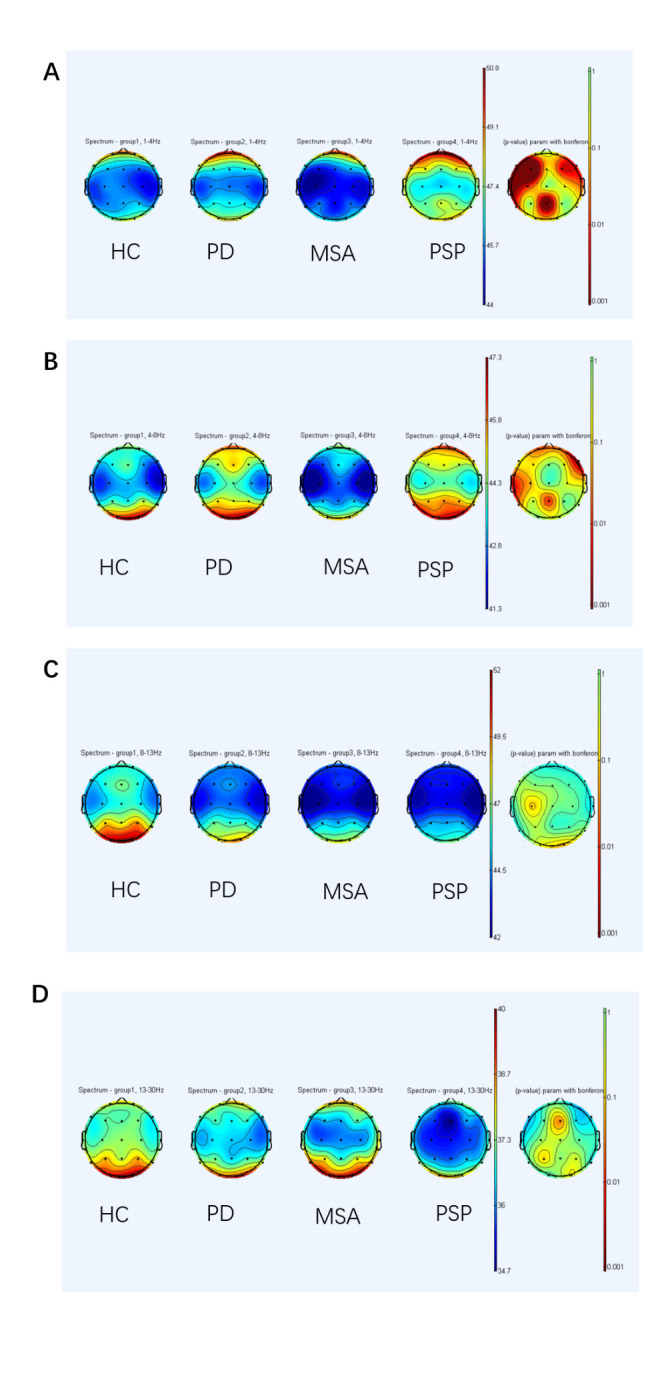
Spectral power of frequency bands shown with topographic maps in different groups. Spectral power in controls, patients with PD, MSA, and PSP, is seen separately. The last column shows the result of Bonferroni's multiple comparisons among groups: *P<0.05. Delta in 2A, Theta in 2B, Alpha in 2C, Beta in 2D. (A) Comparison of delta spectral power in controls, patients with PD, MSA, and PSP reveals a significantly higher power in patients with PSP. (B) Comparison of theta absolute spectral power of specific brain regions presents a higher difference in patients with PD and PSP compared to patients with MSA. (C) The power of alpha does not present a difference among groups. (D) The power of beta does not reveal a difference among groups. PD, Parkinson’s disease; MSA, multiple systems atrophy; PSP, progressive supranuclear palsy

**Figure 3.**
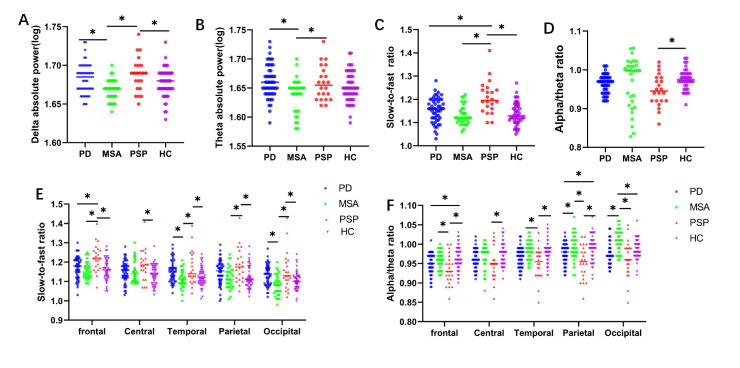
QEEG indices in different groups. (A) Comparison of delta absolute spectral power in patients with PD, MSA, PSP, and controls. (B) Comparison of theta absolute spectral power in different groups. (C) Comparison of slow-to-fast ratios in different groups. (D) Comparison of alpha/theta ratios in different groups. (E) Comparison of slow-to-fast ratios of specific brain regions in different groups. (F) Comparison of alpha/theta ratios of specific brain regions in different groups. *P<0.05, analysis of variance, followed by Bonferroni post hoc tests: PD (N=52), MSA (N=31), PSP (N=22), controls (N=50). QEEG, quantitative electroencephalography PD, Parkinson’s disease; MSA, multiple systems atrophy; PSP, progressive supranuclear palsy

We compared different spectral powers of alpha, theta, delta, and beta bands in patients with PD, MSA, PSP, and HCs. Among the comparisons, the delta and theta powers were significantly higher in patients with PSP, followed by patients with PD and MSA, and HCs (Table 1). Both patients with PD and PSP presented a significant difference in theta and delta ASP compared to those with MSA. However, there was no significant difference among the four groups in the alpha and beta bands in the regional distribution in a topographic map (Fig. 2). Differences in spectral power of the delta (P=0.000, Fig. 3A), theta (P=0.004, Fig. 3B), alpha (P=0.124), and beta bands (P=0.460) among the groups were demonstrated. Considering that age, LEDD, MMSE, UPDRS-III, NMSS, HAMA, HAMD, and H&Y stage might be compounding factors, logistic regression analyses were performed to examine the association of theta or delta power between PD and MSA after adjusting for these factors. The results showed that both delta (p = 0.001) and theta powers (p = 0.002) could independently distinguish between PD and MSA (Table 2).

**Table 1:** Characteristic of Study Population.

Characteristics	PD (N=52) A	MSA (N=31) B	PSP(N=22) C	Controls (N=50) D	P-value
demographics					
Gender, male%	42(80%)	21(68%)	18(81%)	34(68%)	0.131
Age, y	62.1(9.4)	60.2(7.5)	64.5(7.6)	63.9(8.1)	0.120
Clinical features					
duration, y	3.5(3.6)	2.4(1.6)	3.1(2.4)		0.279
LEDD, mg	503.1(124.0)	522.4(133.4)	599.1(152.7)		0.020*
MMSE	24.2(4.06)	25.3(3.7)	21.6(5.5)		0.010*
H&Y stage	2.2(0.7)	2.3(0.7)	2.7(0.7)		0.050
UPDRS-III	21.5(9.6)	22.9(6.4)	28.4(11.5)		0.015*
NMSS (total)	60.7(35.2)	71.4(25.2)	75.7(33.2)		0.122
Cardiovascular	2.3(2.9)	8.1(7.0)	2.7(2.9)		0.000**
Sleep/fatigue	14.0(10.7)	12.9(9.0)	15.1(11.1)		0.746
Mood/apathy	14.3(13.5)	17.7(12.4)	17.0(12.4)		0.462
Perception/illusion	1.1(2.1)	0.3(0.6)	2.1(3.1)		0.013*
Attention/memory	6.1(5.3)	3.9(3.7)	9.6(6.8)		0.001**
Gastrointestinal	6.5(5.7)	6.2(3.2)	8.3(5.1)		0.283
Urinary	6.2(5.3)	8.4(5.0)	9.4(7.7)		0.062
Sexual function	3.6(3.4)	5.5(2.9)	5.0(4.1)		0.048*
Miscellaneous	6.1(5.3)	7.8(2.3)	6.1(5.9)		0.293
HAMA scores	7.9(3.1)	10.7(4.0)	9.0(4.0)		0.004**
HAMD scores	9.2(4.7)	9.6(4.2)	12.9(5.3)		0.009**
QEEG indices					
Global spectral power of frequency (log)					
Delta	1.68(0.02)	1.66(0.01)	1.68(0.02)	1.67(0.01)	0.000 ** A>B (P=0.004) B>C (P=0.002) C>D (P=0.050)
Theta	1.66(0.03)	1.63(0.02)	1.65(0.02)	1.65(0.02)	0.004** A>B (P=0.004) B>C (P=0.043)
Alpha	1.64(0.02)	1.63(0.03)	1.63(0.03)	1.64(0.03)	0.124
Beta	1.57(0.02)	1.57(0.02)	1.56(0.03)	1.57(0.03)	0.460
Slow-to-fast ratio (%) in different regions					
Global	1.15(0.05)	1.13(0.03)	1.2(0.07)	1.13(0.04)	0.000** C>A (0.001) C>B (0.000) C>D (0.000)
Frontal	1.17(0.05)	1.15(0.04)	1.22(0.07)	1.15(0.05)	0.000** C>A (0.003) C>B (0.000) C>D (0.000)
Central	1.15(0.05)	1.15(0.05)	1.18(0.07)	1.13(0.05)	0.010* C>D(P=0.005)
Temporal	1.15(0.05)	1.11(0.04)	1.17(0.08)	1.12(0.04)	0.000** A>B (P=0.008) C>B (P=0.001) C>D (0.010)
Parietal	1.14(0.06)	1.11(0.05)	1.18(0.08)	1.11(0.05)	0.000** C>B (P=0.002) C>D (P=0.000)
Occipital	1.13(0.05)	1.08(0.05)	1.15(0.09)	1.10(0.05)	0.000** A>B (P=0.033) C>B (P=0.005) C>D (0.009)
Alpha-to-theta frequencies ratio (%) in different regions					
Global	0.96(0.02)	0.96(0.06)	0.94(0.03)	0.97(0.02)	0.050 D> C (P=0.035)
Frontal	0.94(0.02)	0.96(0.02)	0.93(0.03)	0.96(0.02)	0.000** D>A (P=0.009) D>C (P=0.000) B>C (P=0.004)
Central	0.96(0.02)	0.96(0.03)	0.94(0.04)	0.97(0.02)	0.011* D>C (P=0.007)
Temporal	0.97(0.02)	0.99(0.03)	0.95(0.04)	0.99(0.03)	0.000** D>C (P=0.000) B>C (P=0.000)
Parietal	0.96(0.02)	0.98(0.02)	0.95(0.04)	0.98(0.02)	0.000** D>A (P=0.033) D>C (P=0.001) B>A (P=0.009) B>C (P=0.000)
Occipital	0.97(0.02)	1.01 (0.02)	0.96(0.05)	0.99(0.02)	0.000** B>A (P=0.000) B>C (P=0.000) B>D (0.011)

Abbreviations: PD, Parkinson’s disease; MSA, multiple system atrophy; PSP, progressive supranuclear palsy; LEDD, levodopa equivalent daily dosage; MMSE, Mini-Mental State Examination; H&Y, modified Hoehn and Yahr staging scale; UPDRS-III, Unified Parkinson's Disease Rating Scale III; NMSS, non-motor symptoms scale; HAMA, Hamilton Anxiety Scale; HAMD, Hamilton Depression Scale; QEEG, quantitative electroencephalography. Results are presented as mean (standard deviation), except for gender. Analysis of variance (ANOVA) and chi-square tests, followed by Bonferroni analysis for normal data. Kruskal-Wallis H analysis is used for non-normal data. Data of clinical features are non-normal data except MMSE and UPDRS-III scores. QEEG indices are normal data. Values with P<0.05 are regarded as significantly related to multiple comparisons.

* P < 0.05.

** P < 0.01.

### Comparison of the slow-to-fast ratio among PD, MSA, PSP and HCs

The global slow-to-fast ratios were compared in the four groups. The results revealed that the slow-to-fast ratio was higher in patients with PSP than in either those with PD or MSA (P=0.000, Fig. 3C). Then, the slow-to-fast ratios of different brain regions were calculated (Fig. 3E). In the frontal region, patients with PD presented significantly lower values than those with PSP (P=0.003). In the temporal and occipital regions, patients with PD presented significantly higher scores than those with MSA. The patients with PSP presented higher scores than those with MSA in almost all regions except for the central region. Logistic regression analyses were conducted to explore the association of the global slow-to-fast ratio (p=0.035) and frontal slow-to-fast ratio (p=0.036) between PD and PSP after adjusting for age, LEDD, MMSE, UPDRS-III, NMSS, HAMA, HMSD, and H&Y stage; the findings revealed that the differences were consistent (Table 2).

### Comparison of alpha/theta ratios among PD, MSA, PSP, and healthy controls

The global alpha/theta ratios were compared in the different groups. However, there was no significant difference among the four groups (P=0.050, Fig. 3D). Then, the alpha/theta ratios of every brain region were calculated. Patients with PD presented significantly lower parietal and occipital regions than those with MSA, while patients with MSA demonstrated significantly higher parietal and occipital regions than those with PSP in almost all brain regions (Fig. 3F). We explored the association of the alpha/theta ratio in parietal (P=0.029, Table 2) and occipital regions (P=0.001, Table 2) between PD and MSA adjusted for compounding factors and revealed the same results.

### Correlations between QEEG indices and H&Y stage, UPDRS-III, MMSE, NMSS, HAMA, and HAMD scores in patients with PD, MSA, and PSP

**Figure 4.**
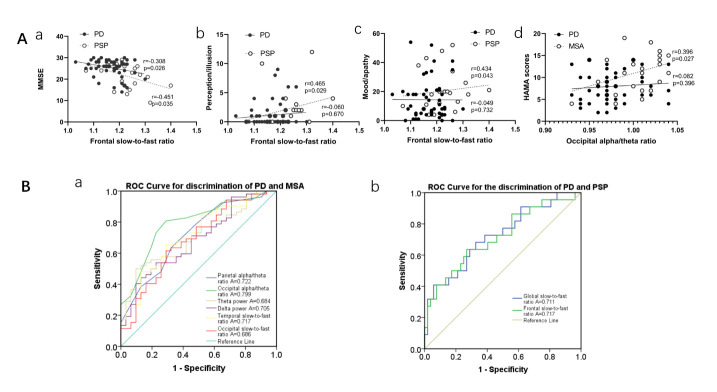
Correlation between QEEG indices and nonmotor dysfunctions, and ROC curves for the evaluation of QEEG for the distinction of PD and MSA/PSP. (A) Relationship between QEEG indices and the severity of nonmotor dysfunctions in PD, PSP, and MSA. Spearman's correlations were used to explore the relationship between QEEG indices and clinical scales in PD(N=52), MSA(N=31), and PSP(N=22). (a) The relationship between the frontal slow-to-fast ratio and MMSE scores in patients with PD and PSP. (b) The relationship between the frontal slow-to-fast ratio and the NMSS perception domain in PD and PSP. (c) The relationship between the frontal slow-to-fast ratio and NMSS mood domain in patients with PD and PSP. (d) The relationship between the occipital alpha/theta ratio and HAMA scores in PD and MSA. (B) ROC curves for the evaluation of QEEG for the discrimination of PD and MSA/PSP. (a) ROC curves for theta spectral power, delta spectral power, parietal alpha/theta ratio, occipital alpha/theta ratio, temporal slow-to-fast ratio, and occipital slow-to-fast ratio to discriminate PD and MSA. (b) ROC curves for the global slow-to-fast ratio and frontal slow-to-fast ratio to discriminate PD and PSP, respectively. QEEG, quantitative electroencephalography PD, Parkinson’s disease; MSA, multiple systems atrophy; PSP, progressive supranuclear palsy; ROC, receiver operating characteristic; MMSE, mini-mental state examination; NMSS, nonmotor symptoms scale, HAMA, Hamilton Anxiety Scale

Correlation analysis of the frontal slow-to-fast ratio and severity of nonmotor dysfunctions revealed a negative correlation (r=-0.308, p=0.026, Fig. 4Aa) with MMSE in patients with PD. Meanwhile, a negative and positive correlation (r=-0.451, P=0.035, Fig. 4Aa) was found with MMSE and NMSS, respectively in the perception (r=0.465, P=0.029, Fig. 4Ab) and mood domain (r=0.434, P=0.043, Fig. 4Ac) in PSP. Meanwhile, the correlation between the occipital alpha/theta ratio and disease severity was examined, and a positive correlation with HAMA scores (r=0.396, P=0.027, Fig. 4Ad) was found in patients with MSA. Details are listed in Supplementary Table 1.

### ROC curves

The ROC curve analysis aimed to distinguish PD from MSA/PSP using the QEEG index (Table 3). In discriminating patients with PD from those with MSA, ROC curves for theta and delta ASP, parietal alpha/theta, occipital alpha/theta, temporal slow-to-fast, and occipital slow-to-fast ratio revealed that the areas under the curves (AUC) were 0.684 [95% confidence interval (CI): 0.568-0.800, p=0.005], 0.705 (95% CI: 0.594-0.816, p=0.002), 0.722 (95% CI: 0.608-0.835, p=0.001), 0.799 (95% CI: 0.701-0.898, p=0.000), 0.717 (95% CI: 0.604-0.829, p=0.001), and 0.686 (95% CI: 0.567-0.805, p=0.002), respectively (Fig. 4Ba). Comparing PD and PSP, the AUC for the global slow-to-fast ratio was 0.711 (P = 0.002), with a sensitivity of 68.2%, a specificity of 67.3%, and a cutoff of 1.178, and the AUC for the frontal slow-to-fast ratio was 0.717 (P = 0.003), with a sensitivity of 63.6%, a specificity of 73.1%, and a cutoff of 1.209 (Fig. 4Bb).

**Table 2:** Binary logistic regression of classification of PD vs MSA/PSP based on separate QEEG indices.

QEEG indices	Unadjusted OR (95%Cl)	Adjusted OR (95%Cl) †	P-value	Adjusted P-value†
PD (N=52) vs MSA (N=31)				
Theta spectral power	0.753(0.628-0.902)	0.672(0.531-0.851)	0.002**	0.001**
Delta spectral power	0.594(0.434-0.814)	0.457(0.296-0.705)	0.001**	0.001**
Parietal alpha/theta ratio	1.377(1.127-1.684)	1.293(1.027-1.626)	0.002**	0.029*
Occipital alpha/theta ratio	1.477(1.224-1.782)	1.485(1.188-1.857)	0.000***	0.001**
Temporal slow-to-fast ratio	0.854(0.771-0.945)	0.873(0.778-0.980)	0.002**	0.021*
Occipital slow-to-fast ratio	0.880(0.804-0.963)	0.895(0.805-0.995)	0.005**	0.040*
PD (N=52) vs PSP (N=22)				
Global slow-to-fast ratio	1.176(1.056-1.309)	1.164(1.011-1.341)	0.003**	0.035*
Frontal slow-to-fast ratio	1.150(1.043-1.267)	1.148(1.009-1.306)	0.005**	0.036*

Binary logistic regression is applied to confirm QEEG indices for PD vs. PSP/MSA after unadjusted or adjusted for confounding variables. † Potential confounding variables of the models are age, sex, H&Y staging, LEDD, MMSE, UPDRS-III scores, HAMA scores, HAMD scores, and NMSS scores.

* p < 0.05,

** p < 0.01,

*** p < 0.001. QEEG, quantitative electroencephalography PD, Parkinson’s disease; MSA, multiple systems atrophy; PSP, progressive supranuclear palsy; LEDD, levodopa equivalent daily dosage; MMSE, Mini-Mental State Examination; H&Y, modified Hoehn and Yahr staging scale; UPDRS-III, Unified Parkinson's Disease Rating Scale III; NMSS, non-motor symptoms scale; HAMA, Hamilton Anxiety Scale; HAMD, Hamilton Depression Scale

### Functional connectivity analysis

**Figure 5.**
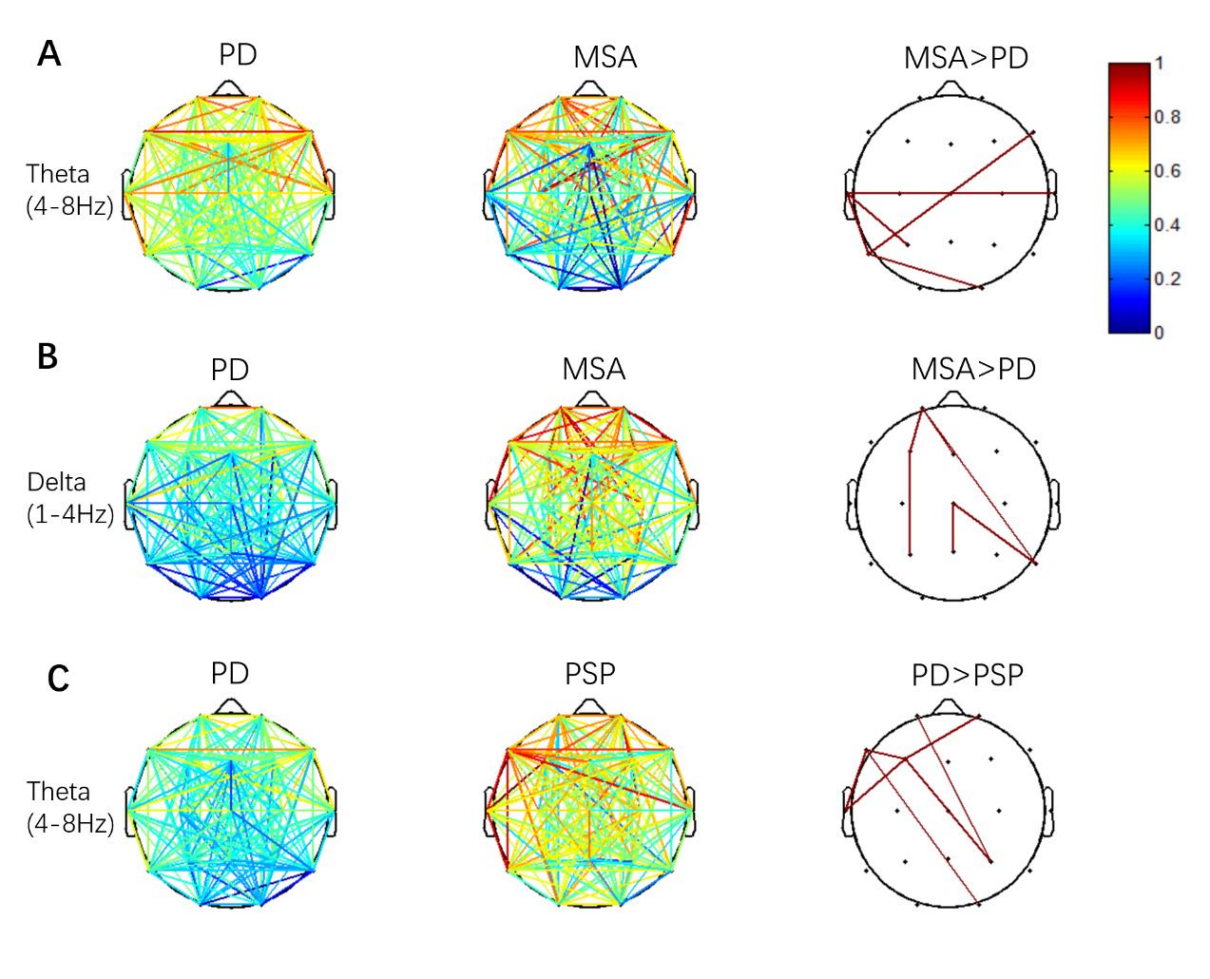
Functional connectivity maps of the PD and MSA/PSP groups in various frequency bands. (A) PD vs. MSA. Patients with MSA show significantly enhanced functional connectivity in theta frequency, especially in posterior regions. (B) PD vs. MSA. Patients with MSA show significantly enhanced functional connectivity in delta frequency, mainly in posterior regions. (C) PD vs. PSP. The patients with PSP show significantly decreased functional connectivity in theta frequency, which is mainly involved in frontal-temporal connections, compared with the patients with PD. The color bar shows the range of changes in the T value during each comparison. The results have been masked with a binary mask of their related p values. The elements with P values greater than 0.05 in the mask matrix are marked as 0, and those with P values less than 0.05 are marked as 1. Pairwise comparisons are conducted by Student’s t-test, and false discovery rate (FDR)-corrected P < 0.05 is considered significant. PD, Parkinson’s disease; MSA, multiple systems atrophy; PSP, progressive supranuclear palsy

Pairwise comparisons of the wPLI were performed to localize differnt functional connections between the PD and MSA/PSP groups. Patients with MSA showed significantly enhanced EEG-FC in theta and delta frequencies, especially in posterior regions than those with PD. Five significantly different functional connections (P3-T3, T3-T4, O2-T5, F8-T5, T3-T5, Fig. 5A) in the theta band and 6 connections (Fp1-F3, F3-P3, Fp1-T6, Fp1-Fz, T6-Cz, Cz-Pz, Fig. 5B) in the delta band were found. Patients with PSP showed significantly decreased EEG-FC in theta frequencies, mainly involved in frontal-temporal connections than those with PD. Seven significantly different functional connections (Fp2-F3, Fp1-P4, F3-P4, F3-F7, O2-F7, F3-F7, F7-T3, Fig. 5C) in the delta band were found.

**Table 3:** ROC curves of QEEG indices for the discrimination of PD and MSA/PSP.

QEEG indices	AUC	Cut-off value	P value	95%Cl	Sensitivity	Specificity
Discrimination of PD (N=52) and MSA (N=31)						
Theta spectral power	0.684	1.654	0.005**	0.568-0.800	0.538	0.774
Delta spectral power	0.705	1.684	0.002**	0.594-0.816	0.500	0.903
Parietal alpha/theta ratio	0.722	0.978	0.001**	0.608-0.835	0.677	0.745
Occipital alpha/theta ratio	0.799	0.989	0.000***	0.701-0.898	0.774	0.804
Temporal slow-to-fast ratio	0.717	1.128	0.001**	0.604-0.829	0.654	0.710
Occipital slow-to-fast ratio	0.686	1.121	0.005**	0.567-0.805	0.615	0.710
Discrimination of PD (N=52) and PSP (N=22)						
Global slow-to-fast ratio	0.711	1.178	0.002**	0.594-0.853	0.682	0.673
Frontal slow-to-fast ratio	0.717	1.209	0.003**	0.584-0.850	0.636	0.731

* p < 0.05,

** p < 0.01,

*** p < 0.001. QEEG, quantitative electroencephalography; PD, Parkinson’s disease; MSA, multiple systems atrophy; PSP, progressive supranuclear palsy; ROC, receiver operating characteristic

## DISCUSSION

In this study, we noted several interesting findings. First, in the same disease duration, patients with PSP revealed a lower MMSE score and higher UPDRS-III and NMSS scores than those with PD, suggesting severe motor and nonmotor functions in patients with PSP than those with PD. Second, the delta and theta ASP were highest in patients with PSP, followed by those with PD and MSA. Third, patients with PD presented significantly higher delta and theta ASP and slow-to-fast ratio in the temporal and occipital regions than those with MSA and a lower alpha/theta ratio in the parietal and occipital regions. Fourth, patients with PD presented a significantly lower slow-to-fast ratio than those with PSP, especially in the frontal region. Fifth, a significant correlation was found between the severity of nonmotor dysfunctions and QEEG indices in PD, MSA, and PSP. Sixth, compared to the FC in patients with PD, FC was lower in the theta band in patients with PSP and more enhanced in the theta and delta bands in patients with MSA; QEEG-related ROC analyses were applied to distinguish PD from MSA/PSP.

An EEG study with transgenic mice demonstrated a close correlation between neuronal accumulation of α-synuclein and slowing of brain oscillations and changes in network excitability [22, 41]. Studies have shown that the complex interaction between cholinergic and other transmitter systems participates in the pathophysiological mechanism of brain electricity slowing and cognitive decline in neurodegenerative diseases [42-46]. Other studies and our results suggest that QEEG is a valuable tool to assess the electrophysiological changes related to cognitive impairment or dementia [47]. Neuropathological evidence suggests that cholinergic degeneration may be an important indicator for early cognitive impairment in PD [48-50]. In a QEEG study, EEG slowing is frequently reported in patients with PD, and delta and theta global absolute powers were found in patients with PDD [51]. Meanwhile, PSP is associated with a widespread cholinergic deficit resulting from a loss of cholinergic neurons, and with an obvious cholinergic deficiency in the cerebral cortex [52]. However, only one study in literature, related to PSP, demonstrated that EEG-slowing was found in six patients with PSP mainly in the frontal region compared to six healthy control participants [53], which was consistent with our results. QEEG signatures reflected the cortical activity and were associated with clinical manifestations, including cognitive impairment, neuropsychiatric disorders, and mental health disorders. QEEG could demonstrate the increased theta power in patients with depression, and the increased alpha power or decreased theta/delta power in patients with anxiety[54]. Moreover, patients with AD with hallucination showed greater delta power compared with those without hallucination [55], while patients with depression with severe delusion were associated with higher regional power in the frontal and temporal regions. Based on previous findings, we proposed that QEEG could be a useful tool to examine cognition and anxiety in PD, PSP, and MSA. Here, we found that patients with PD revealed significantly higher EEG slowing than those with MSA in almost all brain regions (Fig. 3), while patients with PD presented a lower frontal slow-to-fast ratio than those with PSP (Fig. 3E) after adjusting for confounding factors. This finding strongly implies that cholinergic deficits and synaptic dysfunction in PSP/PD are more serious than those in MSA with similar disease duration. These results also suggest that cortical synaptic function is likely deteriorated in patients with PSP, followed by PD and then MSA. Interestingly, we also found that the occipital alpha/theta ratio showed a significant correlation with HAMA scores in MSA, strongly implying that QEEG could be used as a potential indicator to evaluate the severity of anxiety in MSA.

QEEG technology can identify abnormal patterns of cortical activation through power spectrum analysis, and the indicators for EEG-slowing include increasing delta and theta powers, a slow-to-fast ratio, and a lower alpha/theta ratio. It has been documented that, among all indicators for EEG-slowing, the alpha/theta ratio is more sensitive in the early stage of Parkinsonism [56]. Based on our data (Fig. 4), we propose that the EEG features identified in this study are important indicators for cognition in PD and PSP, mood and apathy in PSP, and anxiety in patients with MSA. QEEG indices could contribute to a better understanding of the underlying neurophysiology of nonmotor dysfunctions in PD and PSP/MSA.

Neurodegenerative diseases are usually characterized by a significant reduction in network connectivity, and cortical EEG-FC reflects the intensity of information flow among involved cortical regions [57-59]. Compared with healthy people, patients with PD without cognitive impairment showed a decrease EEG-FC in the alpha and theta frequencies, while those with PD with mild cognitive impairment showed decreased EEG-FC in alpha in posterior distribution, and delta frequency with a generalized distribution [60]. Moreover, several studies have revealed that patients with PDD show reduced EEG-FC in delta band frequency compared to those with PD without dementia [61]. It is suggested that EEG-FC is related to the pathophysiological mechanisms and progression of PD. EEG-FC between PD and atypical parkinsonism was not available in previous studies. An in-depth understanding of QEEG-FC may contribute to understanding the different neuro-pathogenesis of PD and AP.

In the present EEG-FC analysis, significant alterations in EEG-FC were found between PD and MSA/PSP. Previous neuroimaging findings showed that the volume of the striatum and brainstem was significantly reduced in MSA [62]. Moreover, patients with MSA showed bilateral hypometabolism in the basal ganglia through positron emission tomography-computed tomography (PET-CT) [63] examination. In our study, these findings (Fig. 3, 5A, 5B) strongly suggest that MSA presents with less cortical degeneration and might explain the preservation of cognitive function in most MSA cases. Compared to PD, PSP revealed decreased EEG-FC in theta bands, mainly within the frontal-temporal cortex (Fig. 5C), further implying that patients with PSP experience worse cognitive dysfunction and cortical degeneration, which was verified by MMSE scores between PD and PSP (Table 1). Since the disease durations for the three Parkinsonism cases in our study were very similar (Table 1), the QEEG indicators (Figs. 2, 3) and FC network data (Fig. 5) indicated that the cortical neurodegenerative process was likely exacerbated in patients with PSP, followed by PD and then MSA. Meanwhile, PET-CT imaging also showed lower glucose metabolism in the frontal lobe of patients with PSP than those with PD at the early stage [64, 65], which indirectly further verifies our findings. Based on the current results, the decreased EEG-FC and slow-to-fast ratio in the frontal lobe may be promising markers for distinguishing PSP from PD. The changes and differences in EEG-FC between the PD and PSP/MSA groups can be interpreted due to pathophysiological changes, and QEEG indices provide a novel tool to distinguish the underlying neuropathogenesis between PD and PSP/MSA.

To further examine the power of QEEG in discriminating PD from PSP and MSA, ROC was conducted. Our results suggested that QEEG has potential diagnostic value in differentiating PD from PSP (Fig. 4B). Logistic regression analyses (Table 2) were performed to further identify the association of QEEG indices between PD and MSA/PSP after adjusting for the compounding factors. We found that QEEG indices were independent factors differentiating PD from MSA and PSP (Table 2).

There were some limitations in this study. First, the sample size was not sufficiently large, and the relatively small sample size precluded certain subgroup analyses. According to the patient’s initial descriptions and dominant symptoms, PD included tremor-dominant and akinetic/rigid subtypes. Patients with akinetic-rigid are more prone to cognitive decline and depressive symptoms than tremor-dominant patients [66]. Patients without tremors had a higher prevalence of dementia (29%) than those with tremors (14%)[67]. Patients with PD with distinguished subthalamic nucleus FC might explain the various pathophysiological mechanisms in tremor and akinetic/rigid subtypes [68]. Through fMRI, it is proposed that cerebral activity related to Parkinson's tremor first arises in the internal globus pallidus, and then progresses to the cerebello-thalamo-cortical circuit [33]. Another report showed that freezing of gait was associated with a decreased resting-state fMRI-FC within the sensorimotor and default mode networks [69]. Evidently, EEG records the cortical activity and is associated with the non-motor clinical manifestation, such as cognitive impairment, depression, or sleeping disorder. In our study, only a small proportion of patients with PD presented tremor-dominant manifestations. We will enroll more participants in a future study to explore the issue. In addition, future studies with larger sample sizes should investigate whether these EEG markers in PD can predict specific cognitive deficits. Second, the diagnosis of PD, PSP, and MSA was based on the clinical probable diagnosis criteria and was not verified by neuropathological examination. Third, no detailed clinical subtypes were specified for PSP.

## Conclusion

QEEG indicators were fully analyzed in PD, PSP, and MSA, especially the slow-to-fast ratio and alpha/theta ratio in different brain regions, while the differential EEG-FC network using wPLI between PD and PSP/MSA was identified. We observed that the frontal slow-to-fast ratio showed significant positive correlations with cognition in PD and PSP, and with mood/apathy in PSP. The occipital alpha/theta ratio had a positive association with anxiety severity in MSA. Patients with PD presented a lower slow-to-fast ratio and enhanced EEG-FC than those with PSP, especially in the frontal region, and patients with PD revealed significantly higher EEG-slowing and lower EEG-FC than those with MSA. QEEG could be a promising noninvasive tool to understand the pathogenesis of nonmotor dysfunctions in Parkinsonian disorders. These findings shed light on the possible mechanisms underlying the relationships between QEEG indicator activity and nonmotor dysfunctions in PD and AP, notably the involvement of different processes in cortical degeneration. This study indicated that QEEG could be a novel tool to understand the mechanism of Parkinsonism disorders.

## Supplementary Materials

The Supplementary data can be found online at: www.aginganddisease.org/EN/10.14336/AD.2022.0514.

## Data Availability

The data that support the findings of this study are available from the corresponding author, upon reasonable request.
