# Supplementary material for: QEEG Signatures are Associated with Nonmotor Dysfunctions in Parkinson's Disease and Atypical Parkinsonism: An Integrative Analysis
Source: Aging Dis. 2023 Feb 1;14(1):204–18. doi: 10.14336/AD.2022.0514 (PMC9937709; doi:10.14336/AD.2022.0514)
Supplement: Supplementary file 1 — The Supplementary data can be found online at: www.aginganddisease.org/EN/10.14336/AD.2022.0514. [file AD-14-1-204-s.pdf]

SUPPLEMENTARY DATA

QEEG Signatures are Associated with Nonmotor Dysfunctions in Parkinson's Disease and Atypical Parkinsonism: An Integrative Analysis

Hailing Liu[1, 2#], Zifeng Huang[1#], Bin Deng[1#], Zihan Chang[1#], Xiaohua Yang[1], Xingfang Guo[1], Feilan Yuan[1], Qin Yang[1], Liming Wang[3], Haiqiang Zou[4], Mengyan Li[5], Zhaohua Zhu[6], Kunlin Jin[7 *], Qing Wang[1 *]

© 2022. Liu H et al. Published online at http://www.aginganddisease.org/EN/10.14336/AD.2022.014

SUPPLEMENTARY DATA

Supplementary Table 1. Correlation analysis of EEG indices and all variables in PD, PSP and MSA patients.

Variable	Frontal slow-to-fast ratio				Occipital alpha/theta ratio			
	PD		PSP		PD		MSA	
	r	p	r	p	r	p	r	p
H&Y stage	-0.034	0.812	0.154	0.493	0.111	0.438	0.116	0.533
UPDRS-III	-0.039	0.785	0.242	0.278	0.172	0.229	0.183	0.324
MMSE	-0.308	0.026*	-0.451	0.035*	0.005	0.973	-0.001	0.997
NMSS (total)	-0.077	0.588	0.299	0.176	0.156	0.268	-0.094	0.615
Cardiovascular	-0.189	0.180	-0.169	0.452	0.221	0.115	-0.216	0.243
Sleep/fatigue	-0.233	0.096	0.267	0.229	0.090	0.527	0.007	0.969
Mood/apathy	-0.049	0.732	0.434	0.043*	0.141	0.319	0.220	0.235
Perception/illusion	-0.060	0.670	0.465	0.029*	0.087	0.541	0.250	0.175
Attention/memory	-0.189	0.179	0.107	0.634	0.173	0.221	0.078	0.675
Gastrointestinal	0.033	0.816	-0.127	0.572	0.118	0.403	0.117	0.532
Urinary	-0.175	0.214	0.077	0.734	0.051	0.718	-0.161	0.386
Sexual function	-0.187	0.183	0.010	0.966	0.093	0.511	-0.082	0.662
Miscellaneous	-0.217	0.123	0.083	0.081	0.039	0.783	0.225	0.225
HAMA scores	-0.111	0.435	0.108	0.631	0.246	0.082	0.396	0.027*
HAMD scores	0.122	0.391	0.325	0.140	0.070	0.627	0.267	0.147

Abbreviations: r: Spearman's rank correlation coefficient, *P < 0.05

© 2022. Liu H et al. Published online at http://www.aginganddisease.org/EN/10.14336/AD.2022.014
